# Eye Movement Patterns and Approximate Number Sense Task Performance in Williams Syndrome and Down Syndrome: A Developmental Perspective

**DOI:** 10.1007/s10803-019-04110-0

**Published:** 2019-07-02

**Authors:** Jo Van Herwegen, Erica Ranzato, Annette Karmiloff-Smith, Victoria Simms

**Affiliations:** 1 Department of Psychology and Human Development, UCL Institute of Education, 25 Woburn Square, London, WC1H 0AA UK; 2 Department of Psychology, Kingston University London, London, UK; 3 Department of Psychology, Ulster University, Coleraine, UK

**Keywords:** Number development, Williams syndrome, Down syndrome, Eye movements

## Abstract

It has been reported that approximate number sense (ANS) task performance is impaired in individuals with Williams syndrome (WS) and Down syndrome (DS). Research with infants has suggested this impairment is caused by sticky fixation in WS and sustained attention deficits for those with DS. This study examined looking patterns of older children and adults with WS (n = 24) and DS (n = 23) during an ANS task compared to typically developing controls matched for chronological age and those matched for mental age. Results showed that, although there were no group differences, looking patterns changed with chronological age for both the WS and DS groups. Looking behaviour related to ANS performance only in the WS group. Implications for interventions are discussed.

Good mathematical abilities have been shown to rely upon a number of domain specific abilities, such as counting (Passolunghi et al. 2007) and estimation (Halberda et al. 2008), as well as domain general abilities, including inhibition (Cragg and Gilmore 2014), working memory (Passolunghi et al. 2007) and visuo-spatial abilities (Uttal et al. 2013). Studies in infants and young children have indicated that there may be two innate core domain specific systems that underpin mathematical abilities later in life (Feigenson et al. 2004). One core system has been referred to as an object-tracking system that allows rapid estimation of small numbers of objects, referred to as subitizing. In contrast, the approximate number system (ANS) is another core system but this allows rapid estimation of large quantities of objects, sounds, or actions. This system relies upon the ratios presented, known as Weber’s fraction (w). Over development children become better at discriminating between smaller ratios (Halberda and Feigenson 2008). The ANS has been argued to be important for mathematical development later in life, with some evidence that children who have more precise ANS abilities achieving higher performance on mathematical tasks (for recent reviews and meta-analysis see Chen and Li 2014; Fazio et al. 2014). Studies examining children at risk for mathematical difficulties (Costa et al. 2018) and dyscalculia (Mussolin et al. 2010; Mazzocco et al. 2011) have shown that impaired acuity of the ANS contributes to lower calculation skills and mathematical difficulties in general. Although number difficulties are frequently reported in children with neurodevelopmental disorders (Dennis et al. 2009), the contribution of domain general and mathematical specific abilities may differ for each of these disorders.

Williams syndrome (WS) is a rare neurodevelopmental disorder that occurs about 1 in 20,000 live births and is caused by a genetic deletion on the long arm of chromosome 7 (Martens et al. 2008). Cognitively, individuals with WS show IQ scores between 42 and 68 (average = 55) as well as an uneven profile including better language and face recognition abilities, in contrast to their planning and visuo-spatial abilities (Martens et al. 2008). Down syndrome (DS) occurs in about 1 in 800 in life births and is caused by either extra chromosomal material, called trisomy, or, in a small number of cases, by a translocation of genetic material on chromosome 21 (Silverman 2007). Despite the fact that those with DS and WS have similar overall IQ scores (Mervis et al. 2000), individuals with DS show different strengths and weaknesses compared to those with WS, in that they often have poor language and short-term memory abilities, in contrast to their non-verbal abilities (Jarrold et al. 1998).

Previous studies have shown that overall mathematical abilities in individuals with WS and DS are severely impaired and show little development over time (Udwin et al. 1996; O’Hearn and Landau 2007). Yet, the numerical profiles of those with WS and DS differ: those with WS show good verbal counting abilities, even though they often lack an understanding of counting (Ansari et al. 2003; Paterson et al. 2006), whilst those with DS show difficulties with counting (Nye et al. 2001; Sella et al. 2013) and an increased number of counting errors compared to typically developing controls (Porter 1999). Also, mathematical abilities have been shown to relate to different domain general abilities: whilst mathematical abilities develop in line with visuo-spatial abilities in those with DS, this is not the case for those with WS (Ansari et al. 2003; Van Herwegen et al. under review). Finally, there is evidence to suggest that the cause of the mathematical delays in WS and DS can be attributed to different impairments to the different core systems. Infants with WS have been shown to be able to discriminate between small numbers (2 vs 3 dots) but not large sets (8 vs 16) (Van Herwegen et al. 2008). In addition, those with WS have been shown to perform well below typically developing controls on typical ANS tasks in which participants need to indicate which set of dots presented contains a larger amount (Libertus et al. 2014; Rouselle et al. 2013) as well as poor performance on tasks that tap the mental number line (Krajcsi et al. 2009; Opfer and Martens 2012) in which participants have to approximately position a number onto a blank number line. Together, these studies suggest that whilst performance on the object-tracking system that is required for completing tasks with small quantities is age-appropriate in WS, performance on tasks that rely on the ANS is impaired. In comparison, infants with DS have been shown to be proficient at discriminating between large (8 vs 16 dots) but not small amounts (2 vs 3) (Karmiloff-Smith et al. 2012; Camos 2009) and older participants have no difficulties with ANS tasks (Sella et al. 2013) or perform similarly to mental age controls on tasks that are thought to require the mental number line (Abreu-Mendoza and Arias-Trejo 2015; Lanfranchi et al. 2015).

However, the ANS task is also a spatial task in that it requires participants to estimate the total set size of each display and then to say which display is larger. Therefore, attention to the stimuli and switching between the two sets in order to compare their size is an important first contributor to task performance. Although not all dots need to be precisely scanned to estimate the number of objects displayed, eye movements do improve the efficiency of visual processing (Kowler and Steinman 1979). As a result, it has been argued that general basic-level abilities, such as how people scan the displays or move their eyes across the displays, may impact ANS performance and influences the development of number ability (see Van Herwegen and Karmiloff-Smith 2015 for a discussion).

A study by Brown et al. (2003) showed that whilst infants with DS show impaired sustained attention abilities, with fewer periods and less total time of sustained attention compared to control groups, infants with WS performed similarly to TD control groups for sustained attention but showed problems with saccade planning on a double-step saccade planning task. In a double-step saccade planning task infants are shown two targets very briefly and they need to saccade from the first to the second target in order to get a visual reward. In order to successfully saccade to the second target, the infant needs to calculate the second saccade based on the memory of the position of the last target and the vector of the first saccadic movement. Toddlers with WS were unable to combine extra-retinal information with retinal information to the same extent as the other groups, and displayed evidence of other deficits in saccade planning (Brown et al. 2003). In addition, infrared eye tracking methodology has shown that while infants with DS scanned almost the entire array of dots in visual displays during an ANS task, those with WS fixated only on a few of the dots within each display (Karmiloff-Smith et al. 2012). It has been argued that the “sticky fixation” in infants with WS may allow individual dots to be scanned in detail, at the expense of the overall quantity displayed. In contrast, the sustained attention difficulties observed in DS may mean that the majority of items can be scanned due to fast eye movements across the visual scene and this may result in better performance on ANS tasks by those with DS (see Karmiloff-Smith et al. 2012; Van Herwegen and Karmiloff-Smith 2015). Therefore, the difficulties experienced by infants with WS on large number discrimination might be due to differences in looking patterns rather than ANS difficulties per se (Van Herwegen and Karmiloff-Smith 2015; Van Herwegen 2015).

Although previous studies have examined the eye movements of infants with DS and WS during an ANS task, they did not directly compare these to eye movement patterns in TD groups. In addition, the study by Karmiloff-Smith et al. only examined attention patterns in infants and included a very small sample size. It is unclear whether atypical eye movements can still explain impaired performance on the ANS task in older children and adults with WS and DS. A better understanding of whether atypical looking patterns are still be observed in older individuals with WS or DS during mathematical tasks can provide further insight into whether attention patterns or mathematical abilities should be targeted to improve mathematical abilities in WS and DS.

For the first time, the current study examined looking patterns during the ANS task in children and adults with DS and WS compared to a mental age- and chronological age matched control group as well as to examine the relationship between eye movements and chronological age. In addition, we examined how looking patterns related to ANS performance in the different neurodevelopmental disorders. Based upon the previous studies, it was predicted that performance on ANS task would be impaired in both those with DS and WS when compared to TD controls. In addition, if those with WS display sticky fixation during an ANS task, it would be predicted that they would make fewer fixations overall and take a longer time to make a first fixation on a display during the ANS task. They would also have longer first fixation durations and longer fixation durations overall, in contrast to those with DS and TD. As previous studies have shown that those with DS have issues with sustained attention, it was predicted that they would have shorter overall fixation durations and shorter first fixations compared to those with WS and TD on the ANS task.

## Method

### Participants

Twenty-four participants with WS (18 females) aged 8;00 to 51;08 years old (8 children, 10 adolescents and 6 adults) were recruited via the Williams Syndrome Foundation, UK. Due to the rarity of the disorder and fact that participants had to travel to the Research Lab at Kingston University London due to the eye tracker, a large age range was required to allow a reasonable sample size. In addition, a large age range for the WS and DS groups was chosen in order to allow examination of eye movement pattern changes in relation to CA. All WS participants had their diagnosis confirmed either by a genetic test or by clinical diagnosis. Twenty-five participants with DS (11 females) aged 8;08 to 49;02 (7 children, 11 adolescents and 7 adults) were recruited via Down syndrome support groups across the South-East of the UK. All participants with DS had a genetic mutation on chromosome 21 confirmed by parent questionnaire responses. However, for two participants with DS (one child and one adolescent) no eye tracking data could be obtained in more than a third of the trials and thus their data was excluded from the analyses. Twenty-four typically developing (TD) primary school age children (12 females) were recruited whose Raven’s Coloured Progressive Matrices (RCPM; Raven 2007) scores fell within the range of the RCPM scores of the two neurodevelopmental groups (see Farran et al. 2016 for a similar approach). Therefore, the mental age matched (MA) children were much younger than the WS and DS groups (aged between 4;06 and 10;00 years old). Although WS have been shown to display spatial difficulties and show delayed performance on the RCPM task, Van Herwegen et al. (2011) have shown that individuals with WS make the same error patterns as TD children with similar overall scores. Seeing that performance patterns in WS are not atypical, groups can be meaningfully matched on RCPM performance (Van Herwegen et al. 2011). Finally, 24 TD children and adults (17 females) who had similar chronological age to the DS and WS groups (aged 7;11–42;01, including 10 children, 8 adolescents and 6 adults) were recruited. Inclusion of these two control groups allowed us to examine whether eye movements and ANS performance increased in line with chronological age or with mental age abilities and how these relationships differed in the two neurodevelopmental disorder groups. All of the participants had English as a first language and none of the TD participants had a diagnosis for a learning difficulty according to their parental questionnaire. Further participant details can be found in Table 1.

**Table 1:** Participant characteristics and behavioural measures by group, including mean chronological age (CA) in years;months, mean raw score from Raven’s Coloured Progressive Matrices (RCPM) on which mental age (MA) was based, and performance on ANS task

Group	N	Age		RCPM		ANS	
	Count	Mean	*SD*	Mean	*SD*	Mean	SD
Williams syndrome	24	19;04	12;00	15.61	4.57	36.32	5.65
Down syndrome	23	21;01	10;06	16.05	6.05	34.39	7.09
CA controls	24	18;02	8;09	31.56	3.78	42.92	5.60
MA controls	24	6;05	1;08	17.43	6.54	38.75	5.86

### Materials

#### Raven’s Coloured Progressive Matrices

This task measures fluid intelligence and includes 36 trials in which the participant had to identify which picture out of 6 options completed a pattern.

#### ANS Task

**Fig. 1:**
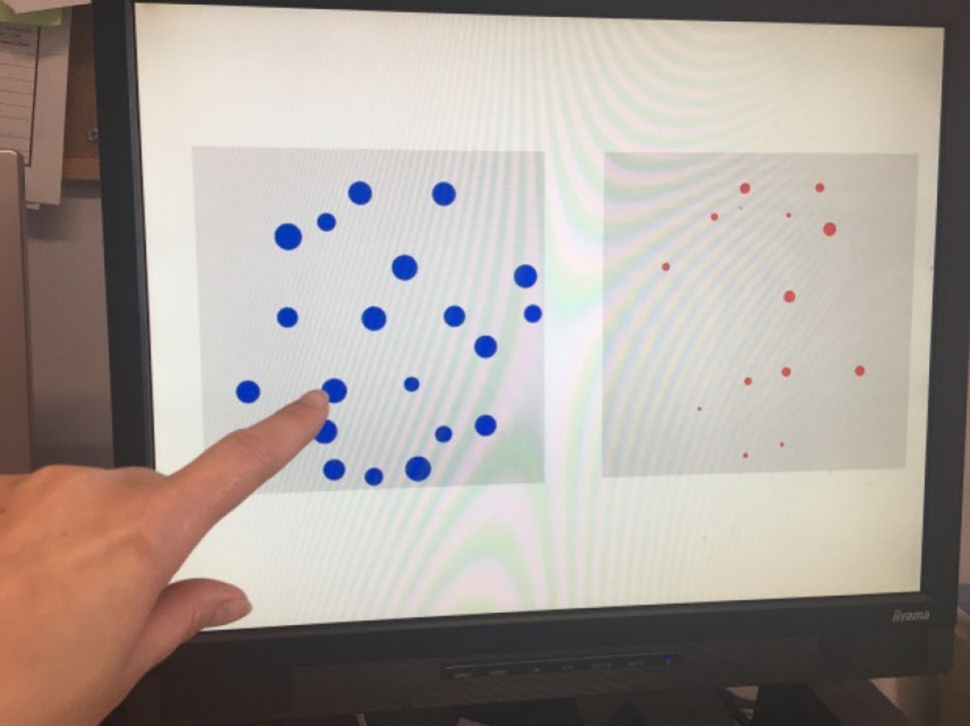
Example of ANS task trial

In this computer task children were presented with a set of dot presentations presented in a white square on the left and right of the screen across 48 trials (see Fig. 1). The dot presentations included between 5 and 28 dots, either red or blue ones, and the dot presentations in each trial included either ratios 0.5, 0.6, 0.7 or 0.8. In half of the trials dot size correlated with the number of dots (i.e., congruent trials) and in the other half of the trials dot size did not correlate with the number of dots (i.e., incongruent trials). The presentation with ‘more’ dots was counterbalanced and appeared on either the left or right side of the screen. Children were asked to select the dot presentation that had ‘more’ by saying the colour of the dots or by pointing at the correct display (see Van Herwegen et al. 2018 for a similar task).

Prior to the actual ANS task, all participants were administered a familiarisation task that assessed whether children understood the concept of ‘more’ and whether they could discriminate and name the colours red and blue. In this familiarisation task children received up to 24 trials (or until they have 8 consecutive trials correct). Each trial showed two dot presentations that had a ratio difference of 1/3 between them. In half of the trials area correlated with number while in the other trials area did not correlate with number. Participants received feedback both when they gave a correct and incorrect answer (see Negen and Sarnecka 2015 for a similar approach).

Eye movements were recorded using a Tobii T120 screen-based eye tracker. The stimuli were presented on a 17–inch monitor by E-prime software version 2.0.10 professional software. Eye-movement recordings were controlled with Tobii’s Studio software (version 2.06) at 120 Hz and fixations were defined using the Tobii I-VT fixation filter.

### Procedure

Parents were provided with detailed information about the project and provided written consent, whilst verbal assent was obtained from all children. This project had received favourable opinion from the Kingston University’s Ethics Committee.

Participants were seated in a windowless room facing the eye tracker monitor at a distance of 60 cm and completed the training task followed by the ANS task. A five-point calibration was conducted before the ANS task started. Participants were instructed to watch the screen and to say the colour of the display that included more dots (red or blue) as fast as they could.

### Data Analysis Plan

For the eye movement data, four different measures were considered: time taken to first fixation, length of the first fixation, total number of fixations, and average fixation duration. These measures were averaged across the left and right display. Square areas of interest were drawn around the each of the displays including an additional 50 pixels on each side to allow for more sensitivity within the data capturing. As trial durations finished as soon as the participant responded or when 1500 ms had elapsed, a proportion score was calculated for the total number of fixations taking into account the trial duration in milliseconds. Thus, this proportion score represents the number of fixations per 1 ms.

Eye movements differences between groups were compared using ANOVAS with group as between factor. Post-hoc differences were examined using Bonferroni comparisons. Greenhouse–Geisser values were reported when sphericity assumptions were violated. Pearson correlations were used to examine relationships between the different eye movement measures and ANS performance within each group.

## Results

### Background and Behavioural Measures

Although the MA children were significantly younger than the WS (*p* < .001) and DS (*p* < .001) groups; *F*(3,94) = 12.602, *p* < .001, η_p_^2^ = .294, there were no significant difference in age between the WS, DS, and CA groups (all *p*s > .05).^1^ There were no significant difference between the MA, WS, and DS groups for RCPM scores; *F*(2,67) = .625, *p* = .539, η_p_^2^ = .019.^2^

For the ANS training task, all participants met the criteria on the practice trials and showed they understood the task (minimum of 8 correct in a row before maximum of 24 test trials were displayed; mean number of trials to get 8 trials correct in a row; CA = 8.00, SD = .000; MA = 8.17, SD = .637; WS = 8.54, SD = 1.141; DS = 9.48, SD = 3.189). For overall performance on ANS task, the CA group performed the best out of all groups but there were no differences between the WS, DS and MA groups; *F*(3,90) = 13.771, *p* < .001, η_p_^2^ = .322, (see Table 1).

As can be seen in Table 2, eye movements were very similar between the four different groups. There were no differences between the four groups for average proportion of fixations; *F*(3,91) = 1658 *p* = .182, η_p_^2^ = .053, for average fixation duration; *F*(3, 92) = .210, *p* < .899, η_p_^2^ = .007, for time to first fixation; *F*(3,92) = 1.880, *p* = .139, η_p_^2^ = .060, or time of the first look; *F*(3,92) = .414, *p* = .744, η_p_^2^ = .014.

**Table 2:** Overview of performance on eye tracking measures for each of the groups: Williams syndrome (WS), Down syndrome (DS), chronological age (CA) controls and mental age (MA) controls

Group	Average proportion of looks		Average fixation duration		Time to first look		Average time of first look	
	Mean	*SD*	Mean	*SD*	Mean	*SD*	Mean	SD
WS	.003	.006	.238	.042	.362	.047	.227	.023
DS	.003	.007	.232	.075	.386	.059	.220	.036
CA controls	.003	.007	.244	.061	.346	.082	.222	.029
MA controls	.003	.007	.244	.067	.388	.085	.230	.044

However, examination of the correlations between chronological age and eye movement patterns in each of the groups revealed interesting group differences. As can be seen in Table 3, chronological age correlated negatively with overall fixation duration and time of first fixation but positively with the average proportion of fixations in the WS group. These results show that with increasing chronological age participants with WS had more fixations that were shorter and also shorter first fixations. This implies that older participants with WS switch their eye movements between the two displays more. For the DS group, there was only a positive correlation for the time to first fixation, showing that with increasing chronological age DS participants take longer to engage with the task. In the control groups, there were only significant correlations with the time of the first fixation variable. However, in the CA controls there was a significant negative correlation, showing that with increasing CA their first look was shorter, whilst in the MA group there was a positive correlation between age and the time of the first fixation.

**Table 3:** Correlations between chronological age and eye tracking measures for each of the groups: Williams syndrome (WS), Down syndrome (DS), chronological age (CA) controls and mental age (MA) controls

Group	Proportion of fixations	Duration of fixations	Time to first fixation	Time of first fixation
WS	.523**	− .530**	− .059	− .498**
DS	− .250	− .320	.418*	− .216
CA controls	− .030	− .099	− .277	− .658**
MA controls	.217	.109	.053	.357*

**p < .001, *p < .05

As can be seen in Table 4, none of the eye movement patterns related to ANS performance in either the CA, MA group, or DS group. However, for the WS group there was a medium sized significant negative correlation; those with shorter average fixation durations had better ANS performance; *r*(22) = − .443, *p* = .039.

**Table 4:** Correlations between ANS Performance and eye tracking measures for each of the groups: Williams syndrome (WS), Down syndrome (DS), chronological age (CA) controls and mental age (MA) controls

Group	Proportion of fixations	Duration of fixations	Time to first fixation	Time of first fixation
WS	.363	− .443*	− .165	− .288
DS	.400	− .150	.234	.065
CA controls	− .113	.054	− .115	.092
MA controls	− .107	.117	− .012	.243

**p < .001, *p < .05

## Discussion

Although previous studies have suggested that impaired mathematical abilities in infants with WS and DS are caused by atypical looking patterns (Karmiloff-Smith et al. 2012; Van Herwegen and Karmiloff-Smith 2015), the current study directly examined for the first time whether atypical looking behaviours can still be observed in older participants with WS and DS, whilst they perform a classic ANS task. In addition, we examined how looking patterns related to chronological age and explored whether looking patterns could explain ANS performance in WS and DS.

Overall, the current results showed no differences in eye movement patterns during the ANS task for either the WS or the DS group compared to CA and MA matched controls. These findings are in contrast to previous studies that have examined eye movement patterns in infants with WS and DS who performed either a double-step paradigm (Brown et al. 2003) or ANS task (Karmiloff-Smith et al. 2012). However, examinations of how eye movements related to chronological age showed some interesting group differences that suggest that eye movement patterns may change with age.

In the WS group, the number of fixations increased with CA whilst the total length of fixations, and the length of the first fixation decreased in line with CA, suggesting that as participants with WS get older they looked differently at the stimuli. For the DS group, there was a significant change for time to first fixation in relation to their CA, in that the older participants took longer to move their attention from the fixation cross to the stimuli. Time to first fixation represents how fast participants engage with the task and start scanning the stimuli. Unlike the WS and CA groups that showed a shorter first fixation over development, indicating that these participants showed faster engagement with the task with increasing age, those with DS show evidence of a disengagement with the task with age. However, it is unclear *why* this is the case. One possibility is that as this DS group get older they have more difficulty in moving their eyes or planning saccades which results in longer times to first fixation. Another possibility is that with age, participants with DS are less motivated to complete the task and thus take longer to engage with the task. However, there were no other differences in terms of task engagement between the DS and the other groups, such as average length of fixations and number of fixations. Therefore, the latter explanation seems less likely. Yet, very little is known about the eye movements in older people with DS and thus more research in this area is required.

The correlations in the MA group differed from those in the CA group. For the MA matched group the speed of engagement with the task decreased with age, whilst the speed of engagement increased with age in the CA control group. The difference between these two findings might be related to the restricted nature of the MA sample in terms of their limited age range (i.e. all primary school children aged between 4;6 and 10 years old). As our MA sample was too small to closely explore developmental effects, future studies should examine how looking behaviours change with CA in younger TD populations. Even though looking patterns in TD populations may change with age, the fact that the CA control group did not significantly differ from the WS and DS group in terms of age range means that correlations between eye movements and age between these groups can still be meaningfully compared.

Examinations of how eye movements related to ANS performance within each group also showed some interesting differences between groups. Although there were no significant correlations between these two variables in the CA, MA, and DS groups, shorter fixation durations did relate to better ANS performance in the WS group. This result may suggest that those who have less “sticky fixation” have better ANS abilities. However, correlations do not provide any insight into cause and effect and thus these results should be interpreted with caution. In addition, although the eye movement data from the WS group seems to suggest that as they get older participants with WS showed less “sticky fixation”, as with increasing chronological age they showed less time to first fixation, shorter fixations, and more fixations, there was no direct evidence that those with WS showed heightened “sticky fixation” in comparison to participants in the other groups.

Together, these eye movement results show that, although at a group level they do not differ from CA and MA groups, eye movements during the ANS task seem to change over development in individuals with DS and WS and some of these changes have been found to relate to ANS performance in the WS group. Specifically, in the WS group, a number of factors suggest that they engage with the stimuli differently as they get older and that this relates to better ANS performance. These findings are in line with infant studies in WS (Brown et al. 2003; Karmiloff-Smith et al. 2012) and the suggestion that differences in basic-level abilities, such as eye movements and attention to stimuli, affect task performance and higher level cognitive development across the lifespan in neurodevelopmental disorders (see Karmiloff-Smith et al. 2012; Van Herwegen and Karmiloff-Smith 2015). Together the findings provide further evidence that neurodevelopmental disorders require a neuroconstructivist approach when examining cognitive development and that similar performance across two developmental disorders can be supported by different developmental pathways (Karmiloff-Smith 2009).

The current study included a number of limitations. First of all, the current study included participants from a wide age range and this large variability could have influenced the results in that the relatively small number of participants and large age range may have masked any group differences. Yet, the descriptive data showed very few differences between the groups. Still, the findings of the cross-sectional data need to be followed-up by longitudinal studies in order to confirm how ANS performance and eye movements change over time (see discussion Van Herwegen et al. 2015). In addition, the current study did not include an independent measure of attention behaviour, for example in the form of a gap-overlap task (Elsabbagh et al. 2013), in order to show whether the changes in eye movements with age reflect a different task approach (top-down) or changes in basic-level eye movement abilities as participants get older (bottom-up). Originally, it was planned to include such a gap-overlap task, but technical difficulties prevented us from including this measure in the study. Furthermore, there was less evidence of any atypical looking in the DS group, especially in relation to sustained attention difficulties. However, the stimuli in the current study were only presented for a very short time in order to prevent counting strategies to be employed to solve the task. Studies in TD adults using free viewing tasks without a time limit have shown that participants produced more saccades in longer trial durations in contrast to shorter trial durations (Paul 2018). Therefore, it is possible that the DS group did not show any increased number of fixations or sustained attention difficulties as the trial duration in the current study was too short. Future studies using longer trial durations or free viewing tasks should examine this further.

More broadly, furthering our understanding of how individuals approach the ANS task has important ramifications for intervention. These specific findings may have clinical implications, in that, in order to support basic quantity processing in early childhood infants with WS might require some attention training that focuses on shifting their eyes, whereas in those with DS sustained attention training might be required. However, for older children and adults with WS and DS there is less evidence of any attention difficulties during the ANS task and thus different evidence-based intervention programmes should be considered to raise mathematical abilities rather than attention training programmes. For example, as previous studies have shown that those with WS have difficulties with ANS (Libertus et al. 2014) and tasks that tap the mental number line (Krajcsi et al. 2009; Opfer and Martens 2012), it might be beneficial to train those with WS directly on the number sense or how numbers relate to each other in order to improve mathematical abilities. In contrast, those with DS show difficulties with counting but not ANS and thus they may benefit more from interventions that target counting and symbolic representations. It has been recognised that training studies can provide further insight into the underlying mechanisms of cognitive development. Therefore, such training studies could in turn provide further evidence that mathematical difficulties in older participants with WS and DS are caused by difficulties with domain specific numerical skills rather than being maintained by atypical eye movement or attention patterns.

## Footnotes

1 Bayesian analysis indicates moderate evidence in support of the null hypothesis, i.e. that there is no group differences in age, BF_01_ = 5.96.

2 No RCPM scores were obtained for the CA adults and thus no comparisons were carried out for the CA adult group with the other groups. Bayesian analysis indicates moderate evidence in support of the null hypothesis, i.e. that there is no group differences in RCPM, BF_01_ = 5.07.

## References

[CR1] Abreu-Mendoza RA, Arias-Trejo N (2015). Numerical and area comparison abilities in Down syndrome. Research in Developmental Disabilities.

[CR2] Ansari D, Donlan C, Thomas M, Ewing S, Karmiloff-Smith A (2003). What makes counting count? Verbal and visuo-spatial contributions to typical and atypical number development. Journal of Experimental Child Psychology.

[CR4] Brown JH, Johnson MH, Paterson SJ, Gilmore R, Longhi E, Karmiloff-Smith A (2003). Spatial representation and attention in toddlers with Williams syndrome and Down syndrome. Neuropsychologia.

[CR5] Camos V (2009). Numerosity discrimination in children with Down syndrome. Developmental Neuropsychology.

[CR6] Chen Q, Li J (2014). Association between individual differences in non-symbolic number acuity and math performance: A meta-analysis. Acta Psychologia.

[CR7] Costa HM, Nicholson B, Donlan C, Van Herwegen J (2018). Low performance on mathematical tasks in preschoolers: The importance of domain-general and domain-specific abilities. Journal of Intellectual Disability Research.

[CR8] Cragg L, Gilmore C (2014). Skills underlying mathematics: The role of executive function skills in the development of mathematics proficiency. Trends in Neuroscience and Education.

[CR9] Dennis M, Berch DB, Mazzocco MM (2009). Mathematical learning disabilities in special populations: Phenotypic variation and cross-disorder comparisons. Developmental Disabilities Research Reviews.

[CR10] Elsabbagh M, Fernandes J, Jane Webb S, Dawson G, Charman T, Johnson MH, The British Autism Study of Infant Siblings Team (2013). Disengagement of visual attention in infancy is associated with emerging autism in toddlerhood. Biological Psychiatry.

[CR11] Farran EK, Formby S, Daniyal F, Holmes T, Van Herwegen J (2016). Route-learning strategies in typical and atypical development; eye tracking reveals atypical landmark selection in Williams syndrome. Journal of Intellectual Disability.

[CR12] Fazio LK, Bailey DH, Thompson CA, Siegler RS (2014). Relations of different types of numerical magnitude representations to each other and to mathematics achievement. Journal of Experimental Child Psychology.

[CR13] Feigenson L, Dehaene S, Spelke ES (2004). Core systems of number. Trends in Cognitive Sciences.

[CR14] Halberda J, Feigenson L (2008). Developmental change in the acuity of the ‘‘number sense”: The approximate number system in 3-, 4-, 5-, and 6-year-olds and adults. Developmental Psychology.

[CR15] Halberda J, Mazzocco MMM, Feigenson L (2008). Individual differences in non-verbal number acuity correlate with maths achievement. Nature.

[CR16] Jarrold C, Baddeley AD, Hewes AK (1998). Verbal and nonverbal abilities in the Williams syndrome phenotype: Evidence for diverging developmental trajectories. Journal of Child Psychology and Psychiatry.

[CR17] Karmiloff-Smith A (2009). The importance of cross-syndrome comparisons: A neuroconstructivist approach. Journal of Intellectual Disability Research.

[CR18] Karmiloff-Smith A, D’Souza D, Dekker TM, Van Herwegen J, Xu F, Rodic M, Ansari D (2012). Genetic and environmental vulnerabilities in children with neurodevelopmental disorders. Proceedings of the National Academy of Sciences.

[CR19] Kowler E, Steinman RM (1979). The effect of expectations on slow oculomotor control. II. Single target displacements. Vision Research.

[CR20] Krajcsi A, Lukacs A, Igacs J, Racsmany M, Pleh C (2009). Numerical abilities in Williams syndrome: Dissociating the analogue magnitude system and verbal retrieval. Journal of Clinical and Experimental Neurospychology.

[CR21] Lanfranchi S, Berteletti I, Torrisi E, Vianello R, Zorzi M (2015). Numerical estimation in individuals with Down syndrome. Research in Developmental Disabilities.

[CR22] Libertus ME, Feigenson L, Halberda J, Landau B (2014). Understanding the mapping between numerical approximation and number words: Evidence from Williams syndrome and typical development. Developmental Science.

[CR23] Martens AM, Wilson SJ, Reutens DC (2008). Research Review: Williams syndrome: A critical review of the cognitive, behavioral, and neuroanatomical phenotype. Journal of Child Psychology and Psychiatry.

[CR24] Mazzocco MMM, Feigenson L, Halberda J (2011). Impaired acuity of the approximate number system underlies mathematical learning disability (dyscalculia). Child Development.

[CR25] Mervis CB, Robinson BF, Bertrand J, Morris CA, Klein-Tasman BP, Armstrong SC (2000). The Williams syndrome cognitive profile. Brain and Cognition.

[CR26] Mussolin C, Mejias S, Noël MP (2010). Symbolic and nonsymbolic number comparison in children with and without dyscalculia. Cognition.

[CR27] Negen J, Sarnecka BW (2015). Is there really a link between exact-number knowledge and approximate number system acuity in young children?. British Journal of Developmental Psychology.

[CR28] Nye J, Fluck M, Buckley S (2001). Counting and cardinal understanding in children with Down syndrome and typically developing children. Down Syndrome Research and Practice.

[CR29] O’Hearn K, Landau B (2007). Mathematical skill in individuals with Williams syndrome: Evidence from a standardized mathematics battery. Brain and Cognition.

[CR30] Opfer JE, Martens MA (2012). Learning without representational change: Development of numerical estimation in individuals with Williams syndrome. Developmental Science.

[CR31] Passolunghi MC, Vercelloni B, Schadee H (2007). The precursors of mathematics learning: Working memory, phonological ability and numerical competence. Cognitive Development.

[CR32] Paterson SJ, Girelli L, Butterworth B, Karmiloff-Smith A (2006). Are numerical impairments syndrome specific? Evidence from Williams syndrome and Down’s syndrome. Journal of Child Psychology and Psychiatry.

[CR33] Paul, J. (2018). Toward a refined model of eye movements in visual enumeration. Unpublished PhD thesis, University of Melbourne.

[CR34] Porter J (1999). Learning to count: A difficult task?. Down Syndrome Research and Practice.

[CR35] Raven J (2007). Coloured progressive matrices.

[CR37] Rouselle L, Dembour G, Noël M (2013). Magnitude representations in Williams syndrome: Differential acuity in time, space and number processing. PLoS ONE.

[CR38] Sella F, Lanfranchi S, Zorzi M (2013). Enumeration skills in Down syndrome. Research in Developmental Disabilities.

[CR39] Silverman W (2007). Down syndrome: Cognitive phenotype. Mental Retardation and Developmental Disabilities.

[CR40] Udwin O, Davies M, Howlin P (1996). A longitudinal study of cognitive and education attainment in Williams syndrome. Developmental Medicine and Child Neurology.

[CR41] Uttal DH, Meadow NG, Tipton E, Hand LL, Alden A, Warren C, Newcombe NS (2013). The malleability of spatial skills: A meta-analysis of training studies. Psychological Bulletin.

[CR42] Van Herwegen J (2015). Williams syndrome and its cognitive profile: The importance of eye movements. Psychology Research and Behavior Management.

[CR43] Van Herwegen J, Ansari D, Xu F, Karmiloff-Smith A (2008). Small and large number processing in infants and toddlers with Williams syndrome. Developmental Science.

[CR44] Van Herwegen J, Costa HM, Nicholson B, Donlan C (2018). Improving number abilities in low performing pre-schoolers: Symbolic versus non-symbolic training programmes. Research in Developmental Disorders.

[CR45] Van Herwegen J, Farran E, Annaz D (2011). Item and error analysis on Raven’s coloured progressive matrices in Williams syndrome. Research in Developmental Disabilities.

[CR46] Van Herwegen J, Karmiloff-Smith A, Cohen-Kadosh R, Dowker A (2015). Genetic developmental disorders and numerical competence across the lifespan. Oxford handbook of numerical cognition.

[CR47] Van Herwegen, J., Ranzato, E., Karmiloff-Smith, A., & Simms, V. (under review). What strategies underpin non-symbolic approximate number system task performance in children? Evidence from eye movement behaviour.

[CR48] Van Herwegen J, Riby DM, Farran EK, Van Herwegen J, Riby DM (2015). Neurodevelopmental disorders: Definitions and issues. Neurodevelopmental disorders: Research challenges and solutions.

